# Good Practice in Health Reporting – Guidelines and Recommendations 2.0: The boards of the German Society for Social Medicine and Prevention and the German Society for Epidemiology have approved this second edition of Good Practice in Health Reporting. *December 2018*

**DOI:** 10.25646/6059

**Published:** 2019-09-04

**Authors:** Dagmar Starke, Günter Tempel, Jeffrey Butler, Anne Starker, Christel Zühlke, Brigitte Borrmann

**Affiliations:** 1 Academy of Public Health in Düsseldorf; 2 Local Public Health Office, Bremen; 3 District of Berlin-Mitte; 4 Robert Koch Institute, Berlin; 5 The Governmental Institute of Public Health of Lower Saxony, Hannover; 6 NRW Centre for Health, Bochum

**Keywords:** GOOD PRACTICE, HEALTH REPORTING, GUIDELINES, RECOMMENDATIONS, PUBLIC HEALTH, GERMANY

## Abstract

Health reporting provides a description of the health of the population, analyses problems and demonstrates areas in which action needs to be taken in health care provision, health promotion and disease prevention. Accordingly, it offers a rational basis for participatory processes and health policy decision-making.

This edition of Good Practice in Health Reporting resulted from a revision of the first edition, which was first published in March 2017. It incorporates contributions from experts, and various institutions and associations from the German federal-state and national levels. This revised edition rose out of the need for continual development in health reporting. In some cases, a number of changes were made so that certain aspects could be defined more clearly; in other cases, changes were made to methodology, for example, in order to accommodate participatory and qualitative approaches.

This publication is aimed at providing people working in health reporting with professional direction and guidance. We welcome your feedback.

## 1. Preface

We are very happy to be able to publish this second edition of Good Practice in Health Reporting. Once again, it has been included as a special issue of the Journal of Health Monitoring. Numerous contributions from experts from the German local, state and national levels as well as from institutions and associations have been incorporated into this publication. We would like to take this opportunity to thank everyone who has been able to contribute. We view the extensive discussions surrounding these contributions and the debate that accompanied the publication of the first edition as continued recognition of the importance of our work.

The significant changes that this edition includes were chiefly implemented to accommodate participatory approaches and qualitative methods. However, we continue to view established nationwide, national and international indicators as the foundation for health reporting as they enable the study of temporal trends and regional/inter-municipal comparisons. Nevertheless, qualitative methods can still be used in health reporting as part of mixed method approaches. We believe that they are particularly suitable for developing questions for study and revealing the complexity of certain problem areas.

This edition of Good Practice in Health Reporting provides an expanded list of criteria. The changes made to the criteria on gender, for example, involve issues such as the importance of conducting comparisons within gender-based groups and of accounting for the societal framework behind gender-based differences.

Good Practice in Health Reporting aims to provide guidance to health officials working at the local, state and national levels and to people working in health reporting in other institutions. It underscores the fact that health reporting is not an end in itself, but that it has a social function that follows the old, yet still relevant, principle of providing ‘data for action’.

Health reporting has been tasked with identifying problem areas and fields in which action needs to be taken. Consequently, it helps promote and safeguard the population’s psychological, social and physical health.

The contributions that we received principally concerned legislation, staffing and financing. Although we understand the request for more details about these topics and would certainly welcome the development of the best possible data pools and facilities, formulating such standards would go beyond the reach of a publication on good practice in health reporting.

Finally, we would like to take this opportunity to wish everyone who uses Good Practice in Health Reporting every success. We hope that this publication provides a positive contribution to further research. Although we will not be opening another formal commenting procedure, we continue to look forward to receiving feedback, and, if necessary, the contributions we receive could result in further revisions of Good Practice in Health Reporting.

## 2. Preamble

Health reporting provides an interpretive description of the population’s health, analyses problems, and highlights areas in need of action.

Due to the misuse of medical statistics and social medicine during National Socialism, health reporting was established relatively recently as a steering instrument in Germany. Importantly, the Ottawa Charter for Health Promotion provides an essential foundation for the development of health reporting. In addition to calls for better integration of different policy fields in order to reduce social inequalities in health, the Ottawa Charter particularly underscores the importance of providing information about the population’s health. In 1987, the Advisory Council for Concerted Action in Health Care (now the Advisory Council on the Assessment of Developments in the Health Care Sector) published a report calling for the establishment of health reporting in Germany with the aim of providing data that could be used for targeted resource allocation. In 2012, the World Health Organization’s Regional Committee for Europe also identified the surveillance of population health and wellbeing as one of ten essential public health operations (EPHOs).

Health reporting has been successfully established in many German federal states and municipalities since the 1980s. This process was strengthened by legislation anchoring health reporting as an official undertaking of the public health service. Since the end of the 1990s, the Robert Koch Institute has conducted health reporting at the federal level in close cooperation with the German Federal Statistical Office.

### 2.1 The aims and tasks of health reporting

Health reporting provides information to politicians and the public about the health, illnesses, health risks and mortality of a spatially and temporally defined population. One of its main tasks is interpreting data from different data sources. As a steering instrument in health policy, health reporting acts as an empirical basis with which to make rationally justifiable policy decisions. Furthermore, it accompanies health policy processes and enables public participation. As such, it is embedded within a particular political discourse. Reporting systems at the local, state and national level are subject to the respective legal and political frameworks.

This means that health reporting:

▶ provides a description of the health of the population. It takes into account the unequal social and regional distribution of health risks and potentials for disease prevention, and demonstrates areas at the national, state and local level where action needs to be taken
▶ accounts for gender, migration and any other living conditions that influence the health of the population or selected population groups
▶ acts as a foundation for the cross-departmental planning of disease prevention, health promotion and care provision, and can be used to evaluate health policy measures
▶ involves the continuous collection of data about the health of a population and identifying possible changes in health at an early stage. Therefore, it can be used to make timely health policy decisions
▶ is not only aimed at experts and decision makers from politics and administration but also at the general public
▶ promotes the process of forming public opinion by providing information and enabling people to participate in drawing up health policy objectives supports the civil society concern of participation

### 2.2 The methodological and theoretical foundations of health reporting

Health reporting requires a broad range of data. Although participative and qualitative methods can also be used to identify relevant topics and issues if they are methodologically justifiable, health reporting should be based on valid and, ideally, uniform standardised data. Moreover, data should be sourced from surveys that have been purposely conducted by the public health service, official statistics and process-generated data from other health system institutions (secondary data). Health reporting should rely on purposely undertaken analyses of these data as well as (international/national/nationwide) coordinated, standardised and routinely prepared indicators. The data holder’s expertise in data collection should be included in the interpretation of results, and, where appropriate, in the formulation of recommendations. Finally, it is essential that the work involved in data collection and the willingness to make these data available for health reporting are appropriately recognised.

In some areas, health reporting needs more data than can be provided by the public health service and secondary data sources alone. In these cases, epidemiological studies and representative health surveys should also be used as they offer additional information about the population’s health, health-related behaviour and health care provision.

Health reporting is typically based on an interdisciplinary approach with epidemiology providing its primary methodological and scientific basis. However, health reporting also incorporates theoretical concepts and empirical findings from the social sciences, medicine, social medicine, medical sociology, health economics, health care research, health system research and health evaluation research as well as other disciplines.

The substantive integration of health reporting into diverse reporting systems, such as social, environmental and educational reporting, is becoming increasingly important. Since there are strong correlations between health and disease and socio-structural factors, health reporting also needs to include data from these reporting systems. Due to significant overlaps and interdependencies that exist between health and social reporting, it is impossible to distinguish strictly between these two fields. As such they can achieve synergy effects in most cases. Nevertheless, the objectives and tasks of health reporting mean that it is essential that this field continues to develop independently and that the work conducted in health reporting is undertaken with the appropriate level of expertise.

Finally, as integrated health reporting is carried out interdisciplinarily, intersectorally and under the involvement of various stakeholders, the field requires specific guidelines to be laid out and the development of good practices.

### 2.3 The foundations, framework and resources needed for health reporting

Health reporting is a complex task that requires detailed knowledge and adequate human, temporal, financial and infrastructural resources. Staff involved in health reporting must be adequately qualified and undergo regular training. The provision of appropriate resources enables high quality, practice-relevant health reporting to be carried out, but also provides staff with an appropriate level of recognition.

### 2.4 Good Practice in Health Reporting

Good Practice in Health Reporting is aimed at providing professional guidance for the production of health reports and highlighting the importance of health reporting as a basis for rational policy-making. One of its focuses is the interpretation of results with regard to their relevance for public health and the basis they provide for health policy decision-making.

In some situations it may be necessary, if not essential, to make exceptions to these guidelines. However, in keeping with good practices, wherever this is done, it needs to be clearly mentioned in the report.

Good Practice in Health Reporting complements Guidelines and Recommendations for Ensuring Good Epidemiological Practice [1] and Good Practice in Secondary Data Analysis [2] by providing additional, albeit central, recommendations for health reporting. However, it also highlights sections of these two documents that contain information that is relevant to planning, preparing and conducting empirical studies and processing, analysing and interpreting the resulting data.

Good Cartographic Practice in Health Care [3] should be referred to for more information about the use of cartography in health reporting. If reports contain findings that can be understood as health-related information (for example, if they contain references to health-promoting behaviour or approaches to tackling health-related risks), the guidelines set out in Good Practice Guidelines for Health Information [4] should also be followed.

## 3. Guidelines and Recommendations

### Guideline 1 (Ethics)

**Health reporting must be carried out in accordance with ethical principles and preserve human dignity and human rights.**

*The objectives of public health ethics should be taken into account in health reporting.*

#### Recommendation 1.1

Results that highlight specific problems among individual population groups should be published with the differentiation and objectivity that is expected of scientific studies.

#### Recommendation 1.2

Health reporting should consider the lives and needs of different social groups and should not be discriminative. This applies to all phases of health reporting.

#### Recommendation 1.3

The indicators used to analyse health-related issues should meet ethical standards. Parameters and indices need to be reviewed to ensure that they are not based on normative assumptions or implicit value judgements.

#### Recommendation 1.4

Health reporting should maintain academic distance and must not provide a voice for interest groups. By providing objective, verifiable information, health reporting creates transparency.

### Guideline 2 (Framework)

**Health reporting requires a defined political and organisational framework and should be anchored in legislation at all policy levels.**

*The legislative basis extended to health reporting should set out the requirements needed to meet scientific quality standards and specify the conditions and framework needed to ensure a good standard of health reporting.*

#### Recommendation 2.1

In addition to sufficient time, financial and infrastructural resources, staff involved in health reporting need appropriate methodological and technical qualifications.

#### Recommendation 2.2

In the case of externally commissioned health reporting, legally binding arrangements should be made for drawing up health reports, accessing and using data, and for supplementary analyses and expertise. This also applies when working together with scientific institutions.

### Guideline 3 (Public Health)

**Health reporting should provide an empirical foundation for health policy decision-making.**

*Health reporting identifies areas from which professionally-based recommendations can be derived. The aim is to improve the health of the population and to take equal opportunities into account.*

#### Recommendation 3.1

Health reporting should focus on and analyse issues that are relevant to public health (such as issues that concern certain population groups or that are related to specific illnesses and clusters of illnesses).

### Guideline 4 (Subject of the Report)

**Health reporting must use data to support its descriptions of current aspects of the health status of the population or population groups. It should provide information and analyse health determinants, frameworks and other health-related aspects.**

*Health reporting involves the study of explicit, operationalisable issues. This provides the basis of a particular design that takes into account the study population, the underlying data, as well as data collection and analysis. As such, estimates can be made of the time and costs associated with reporting and of the scope in which the results can be applied.*

#### Recommendation 4.1

The topicality, public health and policy relevance of an issue should be considered when selecting the topics of health reporting. The aim of a selection, the reason why it was made, and the relevant target groups should all be stated.

#### Recommendation 4.2

When dealing with the issues at the focus of a particular health report, the latest scientific research should be consulted in order to avoid redundancies and outdated hypotheses.

#### Recommendation 4.3

Health reporting should integrate findings from other reporting systems such as social, educational and environmental reporting. This provides an appropriate basis with which to interpret the results and to position them within the literature.

#### Recommendation 4.4

The selection of the population under study and the indicators chosen to represent it should be supported with reference to the issue at hand.

### Guideline 5 (Working Basis)

**Health reporting should be based on the best available data, indicators that have been accepted at different policy levels, as well as the latest research.**

*Health reporting needs access to socio-demographic, socio-structural and regionally differentiated data. Data collection should always be subject to quality assurance.*

#### Recommendation 5.1

A review should be conducted of the relevance, representativeness and informative value of any data that is used. The data owner should be clearly stated.

#### Recommendation 5.2

The selection of the indicators and the literature used to interpret the results should reflect the latest research and consider the entire range of the issue at hand.

#### Recommendation 5.3

Data for the indicators should be collected continuously so that temporal developments can be observed. Regional comparisons of health-related issues should be undertaken using standardised indicators.

#### Recommendation 5.4

Health reporting should highlight any gaps in the data and review new data sources as needed.

### Guideline 6 (Data Processing)

**A detailed plan should be drawn up for the acquisition and storage of all data used in health reporting, for data processing, plausibility testing, coding and data provision.**

*The Guidelines and Recommendations for Ensuring Good Epidemiological Practice, Good Practice in Secondary Data Analysis and Good Cartographic Practice in Health Care apply here.*

#### Recommendation 6.1

The choice of primary data and the rules governing data collection should be documented. It is important to ensure continuity in terms of the rules that apply to data collection, the study population and legal requirements.

#### Recommendation 6.2

If the data have already been prepared, evaluated or published elsewhere, the primary reason for their collection, as well as the regulations that governed data collection and evaluation, should be made clear.

### Guideline 7 (Data Analysis)

**Data analysis needs to be carried out promptly using scientific methods. The raw data that provide the basis of the results should be stored in a fully reproducible manner in accordance with freedom of information laws.**

*The Guidelines and Recommendations for Ensuring Good Epidmiological Practice and Good Practice in Secondary Data Analysis apply here. They particularly apply to the documentation requirements associated with calculating complex indicators and indices.*

#### Recommendation 7.1

Established epidemiological indicators and procedures should be used for data analysis in health reporting.

#### Recommendation 7.2

Analyses should be repeatable; results should be replicable.

#### Recommendation 7.3

Qualitative methods from empirical social research are particularly used in health reporting as part of participatory approaches. Decisions to use qualitative approaches should have a methodological justification; data evaluation should be based on established scientific techniques, such as qualitative content analysis.

### Guideline 8 (Interpretation)

**Health reporting should provide an interpretation of the results.**

*A critical discussion of the methods, data and results in the context of the available evidence should form the basis of any interpretation.*

#### Recommendation 8.1

One of the primary tasks of health reporting is to evaluate the results. This process should not be influenced by personal, political or financial interests.

#### Recommendation 8.2

Results should be described against the background of the latest scientific research. This includes considering health determinants that are essential to the issue in question and illustrating their importance for the development of public health. Alternative interpretations of the results, where relevant, should also be discussed.

#### Recommendation 8.3

Any limitations to the transferability of results to other populations or periods must be set out in the report. If a lack of data prevents certain conclusions from being made, this also needs to be stated. When interpreting temporal developments or trends, it is important to note that the significance of the variables under study and the way in which they are defined are liable to change.

#### Recommendation 8.4

Data interpretation and the formulation of recommendations for action are essential aspects of health reporting. Recommendations for action should be drawn up together with stakeholders from other relevant fields.

### Guideline 9 (Data Protection)

**The applicable data protection regulations should be observed when using data for health reporting.**

*Health data are sensitive, and ethical and legal requirements mean that particular data safeguards must be put in place.*

#### Recommendation 9.1

The responsible data protection officer should be involved in the application of the relevant data protection regulations.

### Guideline 10 (Communications)

**Health reporting is not an end in itself. It competes with other socially relevant issues for the attention of the public.**

*Health reporting should rouse people’s interests. Appropriate media, forms of representation and stylistic elements should be used to achieve this aim.*

#### Recommendation 10.1

Health reporting should use clear, non-discriminatory language that the general population can understand and address target groups appropriately.

#### Recommendation 10.2

Health reporting should use various reporting formats and media that are tailored to the interests and informational habits of the target groups. In addition to print media, the results should be distributed digitally using new media formats; accessibility should be taken into account while doing so.

#### Recommendation 10.3

Health reporting should use attractive and appealing designs to present results. Graphics and illustrations should be used to support any claims. Core results should be highlighted.

#### Recommendation 10.4

Health reporting should use the opportunity to present the results proactively to target groups, expert audiences, relevant stakeholders and the interested public.

### Guideline 11 (Quality Assurance)

**Quality control of all relevant instruments and procedures is essential in health reporting.**

*The most important asset in health reporting is probity, and, subsequently, the trustworthiness of the results. Therefore, quality assurance is an indispensable component of health reporting. The scope of the quality assurance undertaken must be in reasonable relation to the overall costs incurred by health reporting.*

#### Recommendation 11.1

Quality assurance should be conducted during all stages of health reporting. It applies to all of the instruments and procedures employed, ranging from data collection (and the choice of data), data preparation and the use of calculations and interpretations to using data to draw up recommendations.

#### Recommendation 11.2

If secondary data are used, they should be checked for plausibility. Any anomalies and/or systematic errors should be reported to the data-collecting authority.

#### Recommendation 11.3

Specially qualified third parties that were not involved in reporting should participate in quality assurance.

## List of Criteria

### Preliminary Note

The following list identifies the criteria that usually need to be taken into account when producing health reports. The relevance of each criterion, however, depends on the aim, the issue and, therefore, the complexity of a particular report. As such, authors should assess the relevance of these criteria for their particular case. Points that do not appear to be relevant can be ticked as ‘Not applicable’.

**1. Scientific Work**

**Table:**

Scientific standards	Yes	No	Not applicable
The following scientific standards were taken into account during the preparation of the report: › the subject has been clearly delineated › the scope of the report is suitable considering the available material and the focus (there are no redundancies, and unnecessary data have been omitted) › the report is structured logically and each section builds upon the last (the second step results from the first) › the sources of any data or information used are clearly stated › methods are described in detail and are suitable to the data being applied › the results are presented in a structured manner › the results are objective (they are neutral and described with the necessary critical distance) › the results are verifiable (the data are available and the results can be reproduced) › the data and results are scientifically accurate and supported by scientific evidence. Observations and findings are reproduced truthfully › premises and conclusions are made clear › data and results from other publications are cited correctly and scientifically › sources have not been chosen selectively	☐	☐	☐

**2. Reporting System**

**Table:**

**a) Transparency of the contracting authority and author(s)**	**Yes**	**No**	**Not applicable**
The report clearly names the contracting authority.	☐	☐	☐
The report names the authors (including their position and institution, where relevant).	☐	☐	☐
The report makes any conflicts of interest clear.	☐	☐	☐
**b) Planning the report**	**Yes**	**No**	**Not applicable**
With regard to its focus, the report has been compiled in a manner that…			
› is interdisciplinary (it involved cooperation between several scientific disciplines)	☐	☐	☐
› is multi-professional (it involved cooperation between several professional groups)	☐	☐	☐
› is integrative (it involved cooperation between several departments/offices/government agencies)	☐	☐	☐
› includes participation by the study population (for example, during its design, and the determination of requirements)	☐	☐	☐
› involves external experts	☐	☐	☐
A review of the availability of financial and human resources has been conducted.	☐	☐	☐
A schedule has been developed involving all relevant actors.	☐	☐	☐
**c) Structure of the report**	**Yes**	**No**	**Not applicable**
The health report is based on the following structure:			
› a table of contents	☐	☐	☐
› a list of diagrams/tables	☐	☐	☐
› a list of abbreviations	☐	☐	☐
› a preface/introduction	☐	☐	☐
› a summary that states the report’s: contracting authority objectives target audience central findings and recommendations	☐	☐	☐
› a background section or a section explaining the need for the report, and, if relevant, the public health relevance of its focus	☐	☐	☐
› a section describing the data	☐	☐	☐
› a section describing the methods	☐	☐	☐
› a section describing the results	☐	☐	☐
› a section discussing the results	☐	☐	☐
› recommendations (see 7. b-c)	☐	☐	☐
The health report contains a credit note that lists:			
› the authors	☐	☐	☐
› the publisher	☐	☐	☐
› the year of publication	☐	☐	☐
› the place of publication	☐	☐	☐
› the contact person	☐	☐	☐
› the number of copies published	☐	☐	☐
A contact address has been provided.	☐	☐	☐
**d) Funding**	**Yes**	**No**	**Not applicable**
The report clearly states the source of funding:			
› funding is provided from the public budget	☐	☐	☐
› funding is (partly) provided by third-party financing (if so, by whom?)	☐	☐	☐

**3. Style, Layout, Printing and Distribution**

**Table:**

**a) The report uses an understandable and appropriate style**	**Yes**	**No**	**Not applicable**
› The general population can understand the report.	☐	☐	☐
The report…			
› adequately addresses target groups	☐	☐	☐
› avoids jargon wherever possible	☐	☐	☐
› avoids ‘run-on’ and convoluted sentences	☐	☐	☐
› uses active instead of passive formulations	☐	☐	☐
› does not use filler words	☐	☐	☐
› explains abbreviations	☐	☐	☐
› presents the data and indicators in an appropriate graphical form	☐	☐	☐
It would be useful to translate the report into plain language.	☐	☐	☐
**b) Overall layout**	**Yes**	**No**	**Not applicable**
The health report has a clear overall layout.	☐	☐	☐
The health report uses the publisher’s corporate design.	☐	☐	☐
**c) Printing**	**Yes**	**No**	**Not applicable**
The report is available in printed form.	☐	☐	☐
The report includes a distribution list.	☐	☐	☐
Interested parties can order the report (by phone, online, by post, fax).	☐	☐	☐
**d) Distribution**	**Yes**	**No**	**Not applicable**
The report is freely available online.	☐	☐	☐
The report is available online after registration.	☐	☐	☐
The online version of the report meets accessibility requirements.	☐	☐	☐
The online version of the report provides readers with the opportunity to submit questions via a contact form.	☐	☐	☐
The publication of the report was announced through various media channels.	☐	☐	☐
Results are presented proactively to the respective target groups.	☐	☐	☐

**4. Subject of the Report**

**Table:**

**a) Objective**	**Yes**	**No**	**Not applicable**
The report’s objective has been explained clearly and is justified.	☐	☐	☐
The report’s objectives could include: › an analysis of data on morbidity and mortality with respect to a relevant population › an evaluation of health-related measures › a reappraisal of a current situation that endangers (or has endangered) the health of the population › a particular theme as well as an analysis of a specific issue (such as with regard to a specific disease, a specific population group or a cluster of illnesses) › identifying factors that negatively affect public health › providing the basis for policy advice, such as on initiating measures for health promotion › providing conclusions that can be tested empirically			
**b) Population/demographic data**	**Yes**	**No**	**Not applicable**
The population on which the report is based is correctly represented.	☐	☐	☐
Depending on the subject of the report, the following elements could be relevant: › population (average population/population on a specific date) › gender distribution › age distribution › youth to old-age ratio › background of migration/immigrant roots: country of birth (in the case of children and adolescents, their parents’ country of birth) date of immigration to Germany nationality › migration (internal/external) › population projections › birth rate › fertility rate › mortality rate › years of life lost › preventable deaths Note: all significant measured values and their definitions can be found in the health reporting indicator set provided by the Permanent Working Group of the Highest State Health Authorities (Arbeitsgemeinschaft der Obersten Landesgesundheitsbehörden, AOLG).			
**c) Gender**	**Yes**	**No**	**Not applicable**
Data evaluation includes a comparison of gender.	☐	☐	☐
The evaluation of the data is gender-sensitive: comparisons are not only made between the sexes, but differences within gender groups are also taken into account, such as those linked to a person’s social situation, age or background of migration.	☐	☐	☐
Gender stereotypes are avoided when explaining gender-based differences, and, instead, social and political circumstances are considered and theoretical approaches are applied.	☐	☐	☐
**d) Social status**	**Yes**	**No**	**Not applicable**
People’s social status, defined by (school) education, occupation, occupational status and income has been considered.	☐	☐	☐
The data are evaluated separately depending on social status and the results are reviewed in relation to social position and, if necessary, social inequality.	☐	☐	☐
The social structure of a particular territorial unit has been taken into account.	☐	☐	☐
In describing the economic situation of the population under study, the following complementary indicators can be considered: › the proportion of unemployed people/people with no income › the proportion of those receiving unemployment benefit (ALG II) › the proportion of people in marginal employment › the median income › the proportion of single parents › the proportion of children in need as defined by Germany’s Social Code (SGB II)			
e) **Age**	**Yes**	**No**	**Not applicable**
Age groups have been categorised in a manner that is appropriate to the issue at hand.	☐	☐	☐
When different areas are compared, an appropriate form of direct age standardisation is used: › old/new European standard population › standard population of the Federal Republic of Germany in the last available year › local age distribution of the federal state in question	☐	☐	☐
If the available data only cover the standard population, indirect age standardisation is used.	☐	☐	☐
The advantages and disadvantages of using the selected standard population have been made clear.	☐	☐	☐
**f) Stages of life**	**Yes**	**No**	**Not applicable**
Depending on the issue covered by the report, individual stages of life (childhood, adolescence, adulthood, younger/older age) are taken into account.	☐	☐	☐
**g) Migration**	**Yes**	**No**	**Not applicable**
Depending on the issue covered by the report, data on experiences of migration are taken into account (such as country of birth, parents’ country of birth, length of stay, native language, nationality, and residency status).	☐	☐	☐
**h) Inclusion**	**Yes**	**No**	**Not applicable**
Depending on the issue covered by the report, the needs of people with disabilities are appropriately taken into account.	☐	☐	☐
**i) Chronological developments and trends**	**Yes**	**No**	**Not applicable**
Temporal comparisons have been conducted to help identify changes in health over time.	☐	☐	☐
Trends have been projected to help track changes in health over time.			
**j) Regional comparisons**	**Yes**	**No**	**Not applicable**
Comparisons are made using suitable, relevant indicators to help determine regional differences.	☐	☐	☐

**5. Basis of the Data; Data Quality**

**Table:**

**a) Data selection**	**Yes**	**No**	**Not applicable**
Data selection is related to the issue covered by the health report.	☐	☐	☐
The data › are routine data sourced from: official statistics (such as those covering hospital diagnoses, causes of death, the severely disabled, incapacity to work, rehabilitation; and pension statistics or statistics provided by nursing care insurers), registries (such as epidemiological cancer registries, myocardial infarction registries) the census the registry office › are from scientific studies. › are from surveys conducted specifically for the report. › are derived from other data sources/from other data holders.			
A review has been conducted to ensure that the data sources provide an appropriate means of answering the issues at hand.	☐	☐	☐
**b) Accuracy**	**Yes**	**No**	**Not applicable**
The report highlights the possibility of statistical errors and these were taken into account while interpreting the data. Possible errors include: › sampling errors (such as during selection) › distortions created by data collection (for example, due to legislation) › missing values › measurement errors (for example, due to variations in standardised tests) › errors during data processing	☐	☐	☐
**c) Timeliness of the data**	**Yes**	**No**	**Not applicable**
The report uses the latest available data.	☐	☐	☐

**6. Data Evaluation**

**Table:**

**a) Number of cases**	**Yes**	**No**	**Not applicable**
The report specifies the absolute number of cases.	☐	☐	☐
The report specifies the relative number of cases (for example, as the number of cases occurring among 100,000 people).	☐	☐	☐
The report defines the population at risk for each issue (the population among which the cases originated and which is at risk of having the disease).	☐	☐	☐
The report clearly defines the numerator and denominator: the numerator states the number of cases or events and the denominator states the population at risk.	☐	☐	☐
**b) Proportions**	**Yes**	**No**	**Not applicable**
Proportions are stated and provide information about the distribution of health-related events, such as the proportion of cancer mortality in terms of overall mortality.	☐	☐	☐
**c) Rates**	**Yes**	**No**	**Not applicable**
Rates are states that deliver information about the frequency of health-related events, such as contact to a doctor, new cases of illness, births or deaths in relation to the population at risk.	☐	☐	☐
**d) Epidemiological measures**	**Yes**	**No**	**Not applicable**
The report provides a calculation of the following epidemiological measures of disease **frequency:**			
› prevalence/prevalence rate	☐	☐	☐
› incidence/incidence rate	☐	☐	☐
› mortality/mortality rate	☐	☐	☐
› lethality/death rate	☐	☐	☐
The report provides a calculation of the following epidemiological measures of disease **association:**			
› standardised mortality rate (SMR)	☐	☐	☐
› standardised incidence rate (SIR)	☐	☐	☐
› relative risk (RR)	☐	☐	☐
› hazard ratio (HR)	☐	☐	☐
› odds ratio (OR)	☐	☐	☐
The report provides a calculation of the following epidemiological **indicators** of trends:			
› absolute risk difference	☐	☐	☐
› relative risk difference	☐	☐	☐
› attributable risk	☐	☐	☐
› population attributable risk	☐	☐	☐
**e) Health economic considerations**	**Yes**	**No**	**Not applicable**
The report takes into account health economic issues in terms of expenditure, costs and financing.	☐	☐	☐
The report takes into account the following calculations: › the cost of illness, for example, direct and indirect costs › health expenditure calculations, for example, expenditure in public health care according to expenditure type, facility, and cost carrier › operating figures from the public health service, such as the number of staff			
**f) Electronic processing and evaluation**	**Yes**	**No**	**Not applicable**
The data used for health reporting is processed and evaluated electronically and the software that was used to do so is clearly stated.	☐	☐	☐
**g) Evaluation strategies**	**Yes**	**No**	**Not applicable**
All steps undertaken during data processing and data analysis have been described and documented transparently (via a log book, program syntax).	☐	☐	☐
The raw data set has been subjected to a plausibility check.	☐	☐	☐
The plausible raw data set is available in its original form (no newly formed or recoded variables have been added to it).	☐	☐	☐
A review of the results on which the main conclusions are based has been conducted using the dual control principle.	☐	☐	☐
**h) Evaluation of qualitative data**	**Yes**	**No**	**Not applicable**
All steps undertaken during data processing and analysis are described and documented transparently.	☐	☐	☐
A consensual validation of the results has been carried out with all of the project participants (possibly including the participants).	☐	☐	☐
The scope of the results is made clear.	☐	☐	☐

**7. Interpretation, Conclusion and Recommendations**

**Table:**

**a) Mapping problems**	**Yes**	**No**	**Not applicable**
The report substantiates specific problems.	☐	☐	☐
The report maps out problems using objective, deliberative interpretations of the results and discusses alternative explanations.	☐	☐	☐
The report avoids generalisations (especially when using qualitative data).	☐	☐	☐
**b) Recommendations**	**Yes**	**No**	**Not applicable**
The evaluation of the results leads the report to draw up recommendations in need of an urgent response.	☐	☐	☐
In the formulation of these recommendations, critical distance has been maintained in order to prevent interest groups from instrumentalising the results.	☐	☐	☐
The report formulates recommendations with a view to developing possible strategies for hazard prevention/risk reduction.	☐	☐	☐
The report makes recommendations for preventive measures.	☐	☐	☐
The report makes recommendations that include opportunities for health promotion.	☐	☐	☐
The congruence between results and recommendations was taken into account when the recommendations were drawn up.	☐	☐	☐
**c) Evaluation of the implementation of recommendations**	**Yes**	**No**	**Not applicable**
The health reporting framework includes an evaluation of the implementation of the recommendations.	☐	☐	☐
